# Anticipatory solastalgia in the Antipodes: Evidence of future-oriented distress about environmental change in Australia and New Zealand

**DOI:** 10.1016/j.joclim.2025.100415

**Published:** 2025-01-13

**Authors:** Samantha K. Stanley, Omid Ghasemi, Robert M. Ross, John R. Kerr, Mathew D. Marques, Niels G. Mede, Sebastian Berger, Mark Alfano, Neil Levy, Marinus Ferreira, Viktoria Cologna

**Affiliations:** a UNSW Institute for Climate Risk & Response, University of New South Wales; b School of Psychology, University of New South Wales; c School of Medicine and Psychology, The Australian National University; d Department of Philosophy, Macquarie University; e Department of Public Health, University of Otago; f School of Psychology and Public Health, La Trobe University; g Department of Communication and Media Research, University of Zurich; h Institute of Sociology, University Bern; i Uehiro Centre for Practical Ethics, University of Oxford; j Collegium Helveticum, ETH Zurich

**Keywords:** Climate emotions, Solastalgia, Anticipatory emotions, Climate policy, Scale validation, Environmental change

## Abstract

**Introduction:**

Lived experience of negative environmental change can evoke distress called ‘solastalgia’. Worldwide, people are reporting emotional challenges relating to awareness of current and continued environmental decline, even without a direct experience of climate change. Our research examines the measurement of *anticipatory* solastalgia: the experience of present distress about expected environmental change.

**Methods:**

Our preregistered research uses survey samples from Australia (*n* = 1450) and New Zealand (*n* = 1022) to validate a recently developed five-item Anticipatory Solastalgia Scale (the ANSOS). We also measured participants’ experiences of climate emotions, expectations of the increased severity of climate-related weather disasters, and support for climate policy.

**Results:**

The ANSOS fits the data well, is invariant across these two countries, and shows good internal consistency. Supporting convergent validity, the more that participants reported experiencing anticipatory solastalgia, the more intensely they reported feeling negative emotions about climate change. ANSOS scores were higher among those who expected more severe future impacts from climate-related weather disasters, and positively related to support for policies that aim to address climate change.

**Conclusion:**

The study adds further evidence for the validity of the anticipatory solastalgia scale; a measure that is designed to facilitate understanding of people's distress responses to the expectations of, and emotional engagement with, environmental threats as the climate changes.

## 1 Introduction

Research is quickly accelerating to better understand the impacts of anxiety felt in relation to climate change on mental health and wellbeing [1], yet the emotional impacts go beyond anxiety [[2], [3]]. One construct that communicates the complex psychological experience of living through environmental loss is solastalgia. Albrecht [[4], [5]] coined the term by merging the Latin words for solace and pain to capture the distress he observed among Australians living in environments transformed by open-cut coal mines. Residents mourned their environment of times past; homesick despite being at home. Albrecht [4] noted that the distress of solastalgia could be sufficiently severe to threaten mental health.

Although originally recognized following industrial change, solastalgia has been observed following environmental disasters exacerbated by climate change, including coastal erosion [[6], [7]] and bushfire [[8], [9], [10]]. Solastalgia predicts poorer wellbeing following bushfire, even after controlling for objective impacts (e.g., damage to property [9]). Albrecht and colleagues [5] conceptualized solastalgia as a response to *lived experience* of environmental change, influencing previous studies that have centred on communities that have experienced, or are experiencing ongoing, environmental degradation [[6], [7], [8]]. However, lived experience of climate change is not a precondition to experiencing emotional impacts [11].

During interviews with people affected by a severe bushfire season, Stanley et al. [10] identified an *anticipatory* form of solastalgia. Specifically, some participants described a future-oriented fear, sadness, and grief, interpreted as solastalgia in *anticipation* of further environmental loss. The severity of the bushfire season was widely described as likely to be repeated in the future, and with increasing frequency [12]. Participants’ current distress about future environmental change is therefore consistent with the idea that the severe bushfire season was representative of the future of Australia's summers. The same is true of climate change more broadly: while we are currently experiencing its effects (e.g., Australia has already warmed by approximately 1.51 °Celsius since 1910 [13]), the effects are slated to get worse [14].

Investigating anticipatory solastalgia relating to climate change quantitatively, Stanley [15] recently developed an Anticipatory Solastalgia Scale, which we refer to as the ANSOS (i.e., ANticipatory SOlatalgia Scale). Reporting on its validation in the United Kingdom and United States, Stanley found correlations supporting the theoretical link between expectations of environmental decline and experience of anticipatory solastalgia and moderate associations between the ANSOS and the discrete eco-emotions of anger, anxiety, and depression felt in relation to climate change (*r*s between 0.40–0.52), likely reflecting that anticipatory solastalgia incorporates a range of negative emotions.

Many aspects of climate change can evoke negative emotions, such as anxiety about one's personal contribution to environmental problems [16], or anger variously felt towards climate deniers, the inaction of politicians, or the consequences for nature and humanity [17]. The focus of anticipatory solastalgia is both more constrained (by examining responses to expected environmental losses and decline due to climate change), while at the same time capturing a broader emotional response than any single discrete emotion (e.g., anxiety about climate change). After an environmental shock, it may be difficult to disentangle grief, sadness, and worry about unwelcome change. Anticipatory solastalgia can provide a label for this experience of more complex distress in the context of climate change and help us understand these experiences relating to future-oriented concerns.

With climate change threatening to dramatically transform the environment, we examine experiences of the recently developed construct of *anticipatory solastalgia*, defined as distress felt in the present about expected future environmental decline [10,15]. Specifically, we examine whether the ANSOS is a valid and reliable measure in Australia and New Zealand (NZ). These countries both have recent histories of extreme weather events linked to climate change, such as bushfire and floods [12,18]. Additionally, similar to many other places in the world [14], both countries will likely experience increased instances of damaging bushfires and floods as climate change worsens. We preregistered our research questions and hypotheses (https://osf.io/vpjkt), summarised in Table 1, which includes documenting how different demographics (age, gender, region of residence) report anticipatory solastalgia in these countries, as well as the association with political orientation, expectations of increased extreme weather events linked to climate change in future, negative emotions about climate change, and climate policy support.

**Table 1:** Overview of research questions and hypotheses.

**Research questions**	**Preregistered hypotheses**
RQ1: Is the Anticipatory Solastalgia Scale a valid and reliable measure in Australia and NZ?	
RQ2: What is the association between anticipatory solastalgia and age in Australia and NZ?	
RQ3: Are there gender differences in the experience of anticipatory solastalgia in Australia and NZ?	
RQ4: Are there differences in anticipatory solastalgia among those living in urban and rural regions?	
RQ5: What is the association between political orientation and anticipatory solastalgia?	H1: Stronger left-leaning political orientation will be associated with higher endorsement of anticipatory solastalgia.
RQ6: What is the association between anticipatory solastalgia and the expectation of increased extreme weather events due to climate change in future?	H2: Anticipatory solastalgia will be positively associated with the expectation of increased extreme weather events due to climate change in future.
RQ7: What are the associations between anticipatory solastalgia and climate emotions?	H3: Anticipatory solastalgia will be positively associated with negative climate emotions (helpless, angry, guilty, ashamed, depressed, pessimistic).
RQ8: What is the association between anticipatory solastalgia and climate policy support?	H4: Anticipatory solastalgia will be positively associated with support for climate policies.

*Note.* We did not preregister hypotheses for RQ1–4.

## 2 Method

### 2.1 Participants

Participants were a subset of those recruited for the Trust in Science and Science-Related Populism Many Labs project [[19], [20]]. We describe the recruitment approach in detail in the Supplementary Materials. Our sample included 1450 participants in Australia (Ages 18–88 years, *M* = 45.49, *SD*=16.79, 48.1 % women) and 1022 in NZ (Ages 18–91 years, *M* = 45.37, *SD*=16.14, 49.5 % women). Most participants lived in urban areas (78.3 % in Australia, 78.6 % in NZ). The Australian National University Human Research Ethics Committee approved ethical aspects of the study (protocol 2022/506, approved 14 November 2022). Participants provided informed consent before progressing into the survey and consented to their de-identified data being made publicly available.

### 2.2 Measures

#### 2.2.1 Anticipatory solastalgia

We included the ANSOS, first adapted and validated in Stanley [15]. Instructions read: “Please rate the extent you agree or disagree with the following statements relating to your feelings about how the environment will be affected by climate change.” Participants responded to the five items in Table 1 from 1 (strongly disagree) to 7 (strongly agree).

#### 2.2.2 Expected events due to climate change

We asked: “To what extent do you think that climate change will increase the impact of the following weather events in the future?” with six conditions (floods, heatwaves, heavy storms, wildfires, heavy rain, droughts) rated from 1 (not at all) to 5 (very much). We computed a mean score (Australia: α=0.96; NZ: α=0.92).

#### 2.2.4 Climate emotions

Participants were asked: “To what extent does climate change make you feel any of the following?”. They rated nine climate emotions from 1 (not at all) to 5 (very strongly), including seven negative climate emotions that are the focus of our analysis: anxious (accidentally omitted from our preregistration), helpless, angry, guilty, ashamed, depressed, pessimistic.

#### 2.2.5 Policy support

Participants read: “Many countries have introduced policies to reduce carbon emissions and mitigate climate change. This can include the implementation of laws aiming to reduce greenhouse gasses, for example. Please indicate your level of support for the following policies.” They rated five policies, e.g., “raising carbon taxes on gas and fossil fuels or coal” using a response scale of: 1 (not at all), 2 (moderately), 3 (very much), 4 (not applicable). We removed ‘not applicable’ responses, but did not compute a mean policy support score because the internal consistency was poor (Australia: α=0.59; NZ: α=0.54). Instead, we analyzed these item-by-item.

#### 2.2.6 Political orientation

Participants read: “Political orientations are often classified on a left-right spectrum or a liberal-conservative spectrum. Please indicate your political orientation.” They rated their political orientation on a scale from 1 (strongly left-leaning) to 5 (strongly right-leaning). Both samples leaned towards the political right (Australia *M* = 3.46, *SD* = 1.08; NZ *M* = 3.50, *SD* = 1.10).

## 3 Results

Data and R syntax are on the OSF: https://osf.io/fbnzu/. See Supplementary Materials for details on our approach to missing data, which used listwise deletion.

### 3.1 Examination of the ANSOS

We constructed confirmatory factor analysis (CFA) models using Lavaan in R [21] with each of the five ANSOS items set to load onto a single latent variable and we used the maximum likelihood estimation method. We preregistered that we would assess model fit as satisfactory where the comparative fit index (CFI) and Tucker-Lewis index (TLI)≥.90, the root mean square error of approximation (RMSEA)≤.08, and good where the CFI and TLI≥.95, RMSEA≤.06 and standardised root mean square residual (SRMR)≤.08 [22]. The model fit the data well in Australia (χ^2^(5)=12.36, *p*=.030, CFI=1.00, TLI=1.00, RMSEA=0.03, 90 % CI [.01, 0.06], SRMR=0.01) and NZ (χ^2^(5)=9.77, *p*=.082, CFI=1.00, TLI=1.00, RMSEA=0.03, 90 % CI [.00, 0.06], SRMR=0.01). All items showed strong loadings onto the hypothesized anticipatory solastalgia factor, and scale reliability (internal consistency) was good (Table 2). An additional, non-preregistered, analysis demonstrated configural, metric, and scalar invariance across the two samples (Table 3, further details in Supplementary Materials).

**Table 2:** Factor loadings from confirmatory factor analyses performed using the Australian and NZ data.

		Australia	NZ
1.	I am worried that aspects of the environment that I value will be lost	.85	.82
2.	I will be increasingly upset at the way the environment looks	.84	.79
3.	My lifestyle will be threatened by changes to the environment	.77	.72
4.	Unique aspects of nature that make the environment special will be lost forever	.84	.79
5.	I am saddened about the unwelcome change I will see in my landscape	.84	.84

	Cronbach's alpha	.91	.89

*Note*. Factor loadings are fully standardized.

**Table 3:** Measurement invariance on the anticipatory solastalgia scale in Australia and NZ.

Model:	χ^2^(*df*)	CFI	ΔCFI	RMSEA	ΔRMSEA	SRMR	ΔSRMR
Configural	22.12 (10)	.998	–	.031	–	.007	–
Metric	24.08 (14)	.999	.001	.024	−0.007	.010	.003
Scalar	32.62 (18)	.998	−0.001	.026	.002	.013	.003

Both Australian (*M* = 5.24, *SD*=1.24) and NZ (*M* = 5.31, *SD*=1.09) samples reported mean levels of anticipatory solastalgia that were above the theoretical midpoint of the 7-point scale, suggesting a tendency to agree they experienced this phenomenon (see Figure S1 for distributions of responses on each ANSOS item; Table S2 for inter-item correlations).

### 3.2 Associations with anticipatory solastalgia

Anticipatory solastalgia was negatively related to age in each country, indicating that younger participants reported greater anticipatory solastalgia than older participants. Welch's *t*-test did not identify gender differences between Australian men (*M* = 5.22, *SD*=1.24, *n* = 746) and women (*M* = 5.26, *SD*=1.25, *n* = 697), *t*(1433.18) = −0.73, *p*=.465, Cohen's *d*=−0.04. A gender difference occurred in NZ, with women reporting significantly greater anticipatory solastalgia (*M* = 5.40, *SD*=1.00, *n* = 506) than men (*M* = 5.22, *SD*=1.17, *n* = 511), *t*(993.18) = −2.69, *p*=.007, Cohen's *d*=−0.17. There were no significant differences between levels of anticipatory solastalgia among Australians living in urban areas (*M* = 5.21, *SD*=1.23, *n* = 1136) and rural areas (*M* = 5.34, *SD*=1.31, *n* = 314), *t*(476.11) = −1.59, *p*=.112, Cohen's *d*=−0.10, and among New Zealanders living in urban (*M* = 5.33, *SD*=1.10, *n* = 803) or rural areas (*M* = 5.22, *SD*=1.06, *n* = 219), *t*(355.54) = 1.33, *p*=.185, Cohen's *d* = 0.10.

**Fig. 1:**
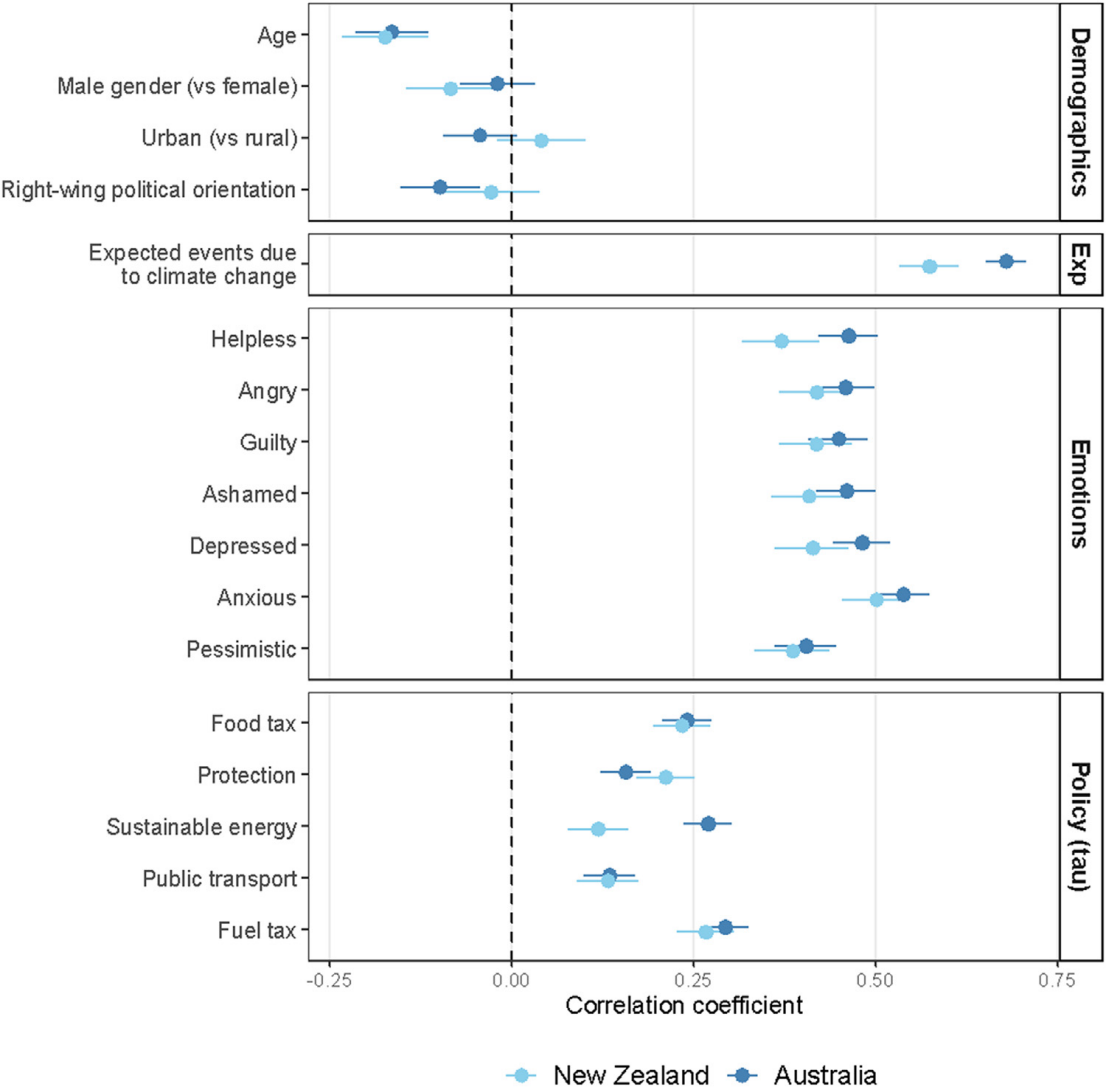
Correlations between anticipatory solastalgia and demographic, emotion, expectation, and policy support variables. *Note.* Correlation coefficients are Pearson's correlations except for policy support, which are Kendall's tau-b correlations. Error bars show 95 % confidence intervals.

Correlations in Table 4 (plotted in Fig. 1) show the hypothesized correlation (H1) between anticipatory solastalgia and a stronger left-wing political orientation only in Australia; there was no significant relationship within the NZ sample. Supporting H2, there was a strong correlation between expected negative weather events due to climate change and ANSOS scores in both countries. Regarding climate emotions, we found support for H3: greater experiences of anticipatory solastalgia correspond to more intense reports of negative emotions in relation to climate change. As we were unable to use a mean score for support for climate policies, we deviated from our preregistration to calculate Kendall's tau-b correlations due to the ordinal nature of these data. Results of these analyses support H4: anticipatory solastalgia was positively related to support for each climate policy. Associations did not substantially change in strength, direction, or significance in our preregistered multiple linear regression analyses controlling for covariates (age, gender, rural/urban residence, education; see Tables S3-S6).

**Table 4:** Associations with scores on the Anticipatory Solastalgia Scale.

	Correlation with ANSOS	
	**Australia**	**NZ**
Age	*r*(1442) = −0.16, *p* < .001	*r*(1017) = −0.17, *p* < .001
Right-wing political orientation	*r*(1234) = −0.10, *p* < .001	*r*(868) = −0.03, *p* = .404
Expected negative weather events	*r*(1446) = 0.68, *p* < .001	*r*(1019) = 0.57, *p* < .001
**Climate emotions**		
Helpless	*r*(1447) = 0.46, *p* < .001	*r*(1020) = 0.37, *p* < .001
Anger	*r*(1446) = 0.46, *p* < .001	*r*(1020) = 0.42, *p* < .001
Guilt	*r*(1446) = 0.45, *p* < .001	*r* (1020) = 0.42, *p* < .001
Ashamed	*r*(1444) = 0.46, *p* < .001	*r*(1019) = 0.41, *p* < .001
Depressed	*r*(1446) = 0.48, *p* < .001	*r*(1020) = 0.41, *p* < .001
Pessimistic	*r*(1448) = 0.41, *p* < .001	*r*(1020) = 0.39, *p* < .001
Anxious	*r*(1446) = 0.54, *p* < .001	*r*(1020) = 0.50, *p* < .001
**Policy support**		
Raising fuel tax	τ = 0.29, *p* < .001, *n* = 1382	τ = 0.27, *p* < .001, *n* = 972
Expanding public transport	τ = 0.13, *p* < .001, *n* = 1317	τ = 0.13, *p* < .001, *n* = 929
Increasing the use of sustainable energy	τ = 0.27, *p* < .001, *n* = 1327	τ = 0.12, *p* < .001, *n* = 932
Protecting forested and land areas	τ = 0.16, *p* < .001, *n* = 1301	τ = 0.21, *p* < .001, *n* = 936
Increasing taxes on carbon intense foods	τ = 0.24, *p* < .001, *n* = 1332	τ = 0.23, *p* < .001, *n* = 938

## 4 Discussion

Our findings support the use of the ANSOS within our Australian and NZ samples. We found evidence supporting the validity of test scores, the internal consistency of the items in the scale, and that the unidimensional model provided good fit to the data. Strong factor loadings suggest that each ANSOS item is a good indicator of the overall construct, and thus reading the scale items can give health professionals and researchers an idea of what people might be experiencing when they have concerns about environmental change. Correlation analyses demonstrated that anticipatory solastalgia is accompanied by a palette of negative climate emotions, higher among those who expect the environment to be subjected to more extreme weather events exacerbated by climate change, and related to greater support for policy solutions to climate change.

Among the correlates studied, we found that anticipatory solastalgia shared the strongest associations with peoples’ expectations about future climate impacts. Specifically, those who believed that climate change will more severely increase the impacts of extreme weather events tended to report greater anticipatory solastalgia. This finding is consistent with Böhm's [23] theorizing that the emotional responses to projected future environmental risks can be even stronger than emotions caused by past environmental consequences. Indeed, people tend to be particularly averse to loss [[24], [25]]. Because anticipatory solastalgia reflects concerns about future environmental losses, it could motivate support for climate reform to mitigate these expected losses, which is a potential explanation for the positive associations between anticipatory solastalgia and support for climate policy.

Our research also contributes evidence on the correlates of anticipatory solastalgia through examining demographic associations. Younger people in both Australia and NZ expressed greater anticipatory solastalgia, consistent with findings from the US though not the UK [15]. This pattern of findings appears to be unique to *anticipatory* solastalgia; solastalgia following lived experience of environmental change is heightened among older ages [7,10], potentially because older adults have lived longer on, and thus developed a greater connection to, the land that is affected. One potential explanation for the differences in age effects is that the expected environmental losses due to climate change will more severely affect younger generations, reflected in higher anticipatory solastalgia. We also identified that NZ women reported higher anticipatory solastalgia than men, but we did not find gender differences in Australia. Stanley [15] found this gender difference in the US, but not in the UK. Gender differences in climate anxiety are similarly inconsistent. When gender associations are found, they identify women as expressing higher intensity emotion (e.g., [[26], [27]]), suggesting there is potentially a gendered element of experiencing (or reporting) heightened distress in relation to environmental concerns that warrants further study. We also found that while the natural environments in urban and rural settings may differ, people may share concerns about impending environmental loss, or the differences in their anticipatory solastalgia may be too small to detect even in our relatively large samples.

In the UK and US, anticipatory solastalgia has been found to be higher among those closer to the left-wing side of left-right political spectrum [15]. Similarly, in the present study we found that left-leaning Australian participants expressed greater anticipatory solastalgia, though we did not find a significant association between political orientation and anticipatory solastalgia in our NZ sample. Whether the null association is due to insufficient power to reliably detect small effect sizes, or that right-wing political adherents in NZ are similarly moved as are those on the political left by shocks to their environment, is worthy of further research. Regardless, it could be particularly interesting for future research to explore the effects of framing climate change in terms of the threat it poses to cherished places, and whether and how such framing differently moves people across the political spectrum.

Our findings also begin to elucidate who is more likely to experience anticipatory solastalgia and what other emotions, expectations, and policy views they are more likely to have. In light of the growing evidence of the different ways climate emotions affect mental health [28], future research can build on ours by exploring the connections between anticipatory solastalgia, mental health, and wellbeing constructs more broadly. Our validation of the ANSOS can facilitate this further research.

Strengths of this research include the use of open science practices (e.g., preregistration, open data and code) and collection of large samples from the general population in each country. As a result, our findings considerably bolster the overall evidence about anticipatory solastalgia, as previous research drew from smaller convenience samples [15] or identified the construct through qualitative interviews [10]. Research is yet to examine test-retest reliability to establish how stable ANSOS scores are across time, and has only begun to investigate its nomological network. Due to the limitations of survey space, we omitted potentially related constructs, such as actual exposure to environmental shocks. Whether having experienced solastalgia from lived experience of environmental change predisposes people to experience anticipatory solastalgia, for example, is unknown. Collecting longitudinal data on anticipatory solastalgia and including a wider range of correlates are both useful future directions to address the limitations of our research, including the reliance on cross-sectional data and correlational methods, as well as the limited set of covariates and correlates included in our survey.

Future research could also build on our findings and use the ANSOS to investigate the drivers of anticipatory solastalgia and the range of consequences associated with the experience. For example, research could explore whether community efforts that successfully protect cherished natural places from projected disasters are associated with reductions in anticipatory solastalgia over time. Another promising avenue for future research is to test interventions that encourage participants to imagine such losses – before they occur – in case this encourages climate action to protect these areas.

Generalizing about the extent to which anticipatory solastalgia is a shared experience will require research with greater diversity in our samples. There is a growing recognition that climate emotions are experienced around the world (e.g., [29]); however, anticipatory solastalgia research is currently limited to four relatively wealthy, English-speaking nations, precluding cross-cultural analysis. Further research may seek to identify the extent to which anticipatory solastalgia features in other cultures, and if it does, what similarities and differences exist. This future research ought to include participants both within and across a larger range of countries, and a focus on how Indigenous groups experience solastalgia [30]. Building on our research, which highlights the relevance of peoples’ expectations of weather-related impacts of climate change, future research could explore more directly the ways in which people expect climate change to impact national treasures and unique, cherished aspects of the natural environment in their country, and the extent to which such views relate to anticipatory solastalgia, efforts to protect the environment, and support for environmental protection policy.

## Funding

Data collection for this project was supported by funds provided to Samantha K. Stanley from the ANU Research School of Psychology; by funding awarded to Mathew D. Marques under the La Trobe School of Psychology and Public Health Internal Grant Scheme 2022; and by Australian Research Council Discovery Project grant funding awarded to Mark Alfano (DP190101507) and Neil Levy (DP180102384). Samantha K. Stanley is the recipient of an Australian Research Council Discovery Early Career Award (DE240100001) funded by the 10.13039/100015539Australian Government. Neil Levy, Robert M. Ross, and Mark Alfano are supported by funding from the 10.13039/100000925John Templeton Foundation (#62631: NL, RMR, MA; #61378: MA). Viktoria Cologna acknowledges support from the Early-career Fellowship, Collegium Helveticum, ETH Zurich.

## Data availability

Data and R syntax are on the OSF: https://osf.io/fbnzu/.

## CRediT authorship contribution statement

**Samantha K. Stanley:** Writing – review & editing, Writing – original draft, Visualization, Project administration, Methodology, Investigation, Funding acquisition, Formal analysis, Data curation, Conceptualization. **Omid Ghasemi:** Writing – review & editing, Visualization, Investigation, Formal analysis, Data curation. **Robert M. Ross:** Writing – review & editing, Project administration, Investigation. **John R. Kerr:** Writing – review & editing, Visualization, Investigation, Formal analysis, Data curation. **Mathew D. Marques:** Writing – review & editing, Investigation, Funding acquisition. **Niels G. Mede:** Writing – review & editing, Supervision, Project administration, Methodology, Investigation. **Sebastian Berger:** Writing – review & editing, Investigation. **Mark Alfano:** Writing – review & editing, Investigation, Funding acquisition. **Neil Levy:** Writing – review & editing, Investigation, Funding acquisition. **Marinus Ferreira:** Writing – review & editing, Investigation. **Viktoria Cologna:** Writing – review & editing, Supervision, Project administration, Methodology, Investigation.

## Declaration of competing interest

The authors declare that they have no known competing financial interests or personal relationships that could have appeared to influence the work reported in this paper.

## Appendix Supplementary materials

Image, application 1

## Footnotes

Supplementary material associated with this article can be found in the online version at doi:10.1016/j.joclim.2025.100415.
