# Supplementary material for: Anticipatory solastalgia in the Antipodes: Evidence of future-oriented distress about environmental change in Australia and New Zealand
Source: J Clim Chang Health. 2025 Jan 13;23:100415. doi: 10.1016/j.joclim.2025.100415 (PMC12851072; doi:10.1016/j.joclim.2025.100415)
Supplement: Supplementary file 1 [file mmc1.docx]

**Supplementary Materials**

**Recruitment approach**

The study employed a team science approach by recruiting collaborators across the world to sign up to collect data in their country. We engaged Bilendi & Respondi to collect data in Australia and New Zealand using quota sampling to recruit approximately equal numbers of men and women in specified age groups (18-29, 30-39, 40-49, 50-59, 60+ years). All participants completed the same core survey of around 15 minutes, which included demographic items and questions about their access to science information, their opinions on science and scientists, and the measures described below for climate emotions and expected events due to climate change. Key findings from the core survey are reported elsewhere (Cologna et al., 2025). At the end of the core survey, participants were randomized to complete either our items or other measures for another project (which will be reported elsewhere). The Australian and New Zealand data were collected between 25 January and 11 February 2023. Our sample consisted of 1450 Australia-based participants and 1022 New Zealand-based participants who completed the measures for our study and passed preregistered data quality checks^[[1]](#footnote-1)^. These samples were more than sufficient in size to meet common rules of thumb for confirmatory factor analysis (e.g., N > 200, or that sample sizes of 300 are good, 500 very good, etc.; for a review, see Kyriazos, 2018), and to detect small correlations (*r* ~ .07 in Australia, .09 in NZ) based on calculations in G*Power using power of .8 and a critical alpha of .05 (Faul et al., 2009).

Our Australian sample was aged 18-88 years (*M* = 45.49, *SD* = 16.79), 48.1% of the sample identified as women and 51.4% as men (0.3% preferred to self-describe, and 0.2% preferred not to say or did not respond). The majority (78.3%) lived in urban areas (21.7% in rural areas). The most common response regarding their highest completed level of education was tertiary-level (68.6%), followed by secondary (29.9%), with few participants recording primary (1.5%) or no formal education (0.1%). The median household income was AUD87,000.

Our New Zealand sample was aged 18-91 years (*M* = 45.37, *SD* = 16.14), and 49.5% identified as women, 50.0% as men (0.2% preferred to self-describe, and 0.3% preferred not to say or did not answer). Most (78.6%) lived in urban areas (21.4% rural). Educational attainment was most commonly reported at tertiary level (70.7%), followed by secondary (26.9%), and very few at primary level (2.3%; no respondents reported no formal education). The median household income was NZD100,000 (approx. AUD91,2066, using the February 2023 exchange rate).

**Approach to missing data**

There were no missing data on the ANSOS because we used the forced choice setting in Qualtrics. By contrast, for consistency with the larger TISP project, we used the request response setting for other questions, including expected events due to climate change (resulting in missing data of *n* = 1 in NZ, *n* = 2 in Australia), climate emotions (resulting in between 0-4 and 0-1 missing cases per emotion in Australia and NZ, respectively), and policy support (0-2 and 0-1 missing cases per policy in Australia and NZ, respectively). We also removed the ‘not applicable’ responses on the policy support measure, thus total missingness ranged from 68-149 cases in Australia, and 50-93 cases in NZ. Participants could select ‘don’t know’ or skip this question; in both cases, these responses were treated as missing data (*n* = 214 in Australia, *n* = 152 in NZ). Consistent with our preregistered plan, we used listwise deletion to remove participants with missing data in analyses with each variable.

**Measurement invariance**

We conducted measurement invariance analyses to determine whether our Australia and New Zealand-based samples responded similarly to the Anticipatory Solastalgia Scale. This analysis was not preregistered. We used Chen’s (2007) guidelines for evaluating metric and scalar invariance based on unequal sample sizes, given the Australian sample was larger than the New Zealand sample. Assessment of the configural model relies on the same model fit criteria listed in the main text. Metric and scalar invariance are supported if changes in CFI are less than or equal to .005, and changes in RMSEA are less than or equal to .010. For changes in the SRMR values, those less than or equal to .025 are evidence of metric invariance, and .005 or lower for scalar invariance. As shown in Table 1 in the main text, the configural model provided excellent fit to the data. Adding the constraints of metric invariance and scalar invariance resulted in fit changes below the criteria, thus supporting the invariance of the scale across these two samples.

**Figure S1.**

*Distribution of responses on anticipatory solastalgia scale items.*

*
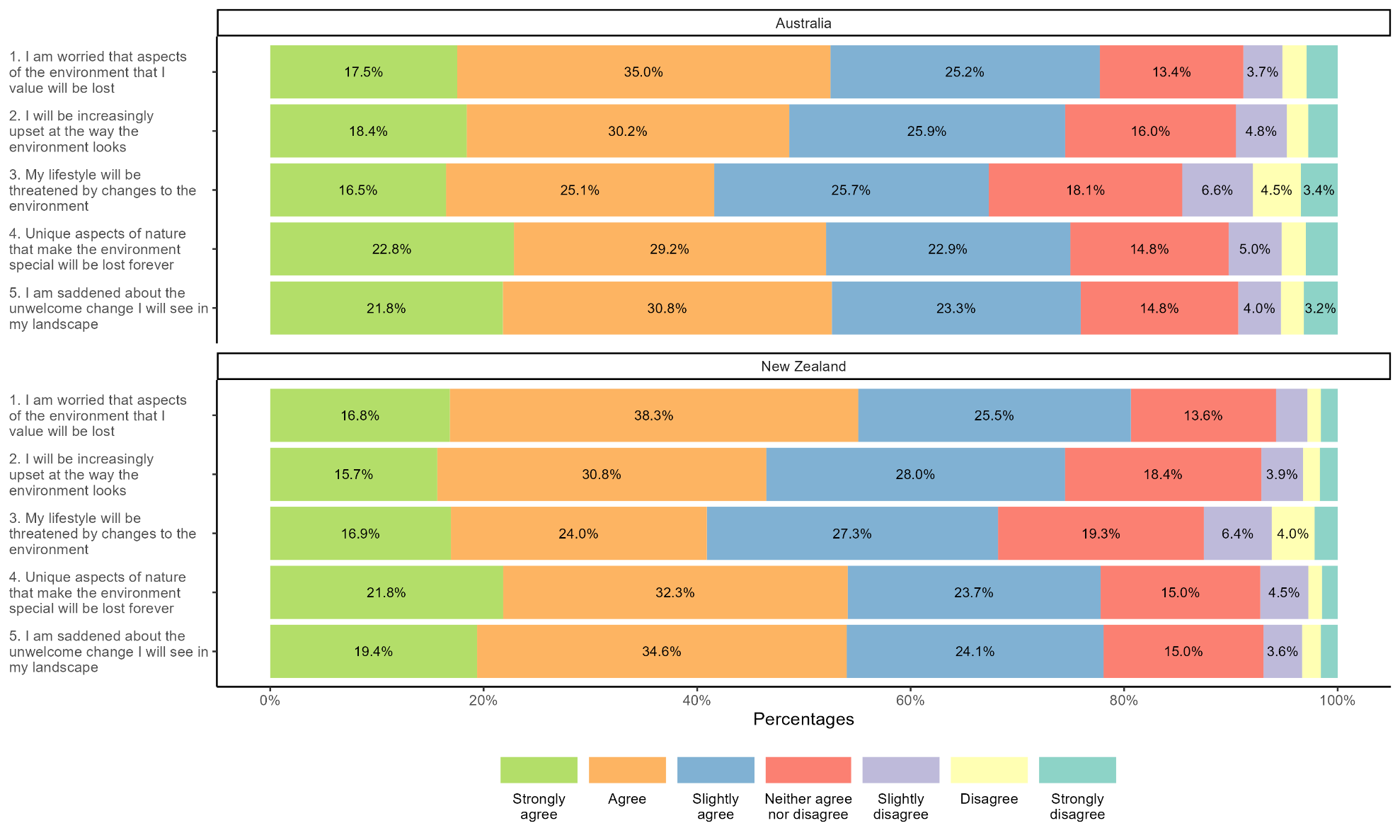
*

**Table S2.**

*Inter item correlations on the anticipatory solastalgia scale in Australia (above the diagonal) and New Zealand (below the diagonal).*

|  | 1. | 2. | 3. | 4. | 5. |
| --- | --- | --- | --- | --- | --- |
| 1. I am worried that aspects of the environment that I value will be lost | - | .72 | .65 | .72 | .71 |
| 2. I will be increasingly upset at the way the environment looks | .66 | - | .64 | .70 | .70 |
| 3. My lifestyle will be threatened by changes to the environment | .59 | .55 | - | .63 | .67 |
| 4. Unique aspects of nature that make the environment special will be lost forever | .66 | .63 | .55 | - | .71 |
| 5. I am saddened about the unwelcome change I will see in my landscape | .68 | .66 | .62 | .66 | - |

*Note.* All correlations in Table S2 are significant at the *p* < .001 level. There was no missing data; sample size for all associations is 1450 (Australia) and 1022 (NZ).

**Table S3.**

*Political orientation as a predictor of anticipatory solastalgia, while controlling for demographics.*

|  | Australia | New Zealand |
| --- | --- | --- |
| Age | -.16*** | -.17*** |
| Gender | -.02 | -.11** |
| Residence (1 = rural, 2 = urban) | -.03 | .05 |
| Education (0 = secondary or lower, 1 = tertiary) | .05 | .09** |
| Political orientation | -.09** | -.03 |
| R^2^ | .04 | .06 |

*Note*. Regression coefficients are standardized beta weights, ***p* < .01, ****p* < .001.

**Table S4.**

*Expected weather events as a predictor of anticipatory solastalgia, while controlling for demographics.*

|  | Australia | New Zealand |
| --- | --- | --- |
| Age | -.02 | -.13*** |
| Gender | .05** | -.003 |
| Residence (1 = rural, 2 = urban) | -.02 | .02 |
| Education (1 = secondary or lower, 2 = tertiary) | .03 | .09*** |
| Expected increase to extreme weather events | .68*** | .56*** |
| R^2^ | .47 | .36 |

*Note*. Regression coefficients are standardized beta weights, ***p* < .01, ****p* < .001.

.

**Table S5.**

*Anticipatory solastalgia as a predictor of climate emotions, while controlling for demographics.*

| **Australia** | Helpless | Angry | Guilty | Ashamed | Depressed | Pessimistic | Anxious |
| --- | --- | --- | --- | --- | --- | --- | --- |
| Age | -.20*** | -.12*** | -.25*** | -.21*** | -.20*** | -.12*** | -.17*** |
| Gender | .07** | .08*** | .08*** | .10*** | .12*** | .10*** | .07** |
| Residence (1 = rural, 2 = urban) | .03 | .002 | .04 | .01 | .02 | .03 | .03 |
| Education (1 = secondary or lower, 2 = tertiary) | .01 | .03 | .07** | .04 | .06** | .08** | .02 |
| Anticipatory solastalgia | .43*** | .44*** | .41*** | .42*** | .45*** | .38*** | .51*** |
| R^2^ | .26 | .23 | .29 | .27 | .30 | .20 | .33 |
| **New Zealand** | Helpless | Angry | Guilty | Ashamed | Depressed | Pessimistic | Anxious |
| Age | -.16*** | -.11*** | -.24*** | -.17*** | -.18*** | -.13*** | -.17*** |
| Gender | .01 | .06* | .03 | .02 | .04 | .07* | .03 |
| Residence (1 = rural, 2 = urban) | -.03 | -.05 | -.04 | -.08** | -.10*** | -.04 | -.03 |
| Education (1 = secondary or lower, 2 = tertiary) | .04 | .10*** | .13*** | .08** | .14*** | .10*** | .05 |
| Anticipatory solastalgia | .34*** | .39*** | .36*** | .37*** | .37*** | .36*** | .47*** |
| R^2^ | .17 | .20 | .25 | .21 | .23 | .18 | .28 |

**Table S6.**

*Anticipatory solastalgia as a predictor of climate policy support, while controlling for demographics.*

| **Australia** | **Raising fuel tax** | **Expanding public transport** | **Increasing use of sustainable energy** | **Protecting forested and land areas** | **Increasing taxes on carbon intense foods** |
| --- | --- | --- | --- | --- | --- |
| Age | 0.99 *** | 1.00 | 1.00 | 1.01 ** | 0.98 *** |
| Gender | 1.51 *** | 0.98 | 0.79 ** | 0.76 *** | 1.54 *** |
| Residence (1 = rural, 2 = urban) | 1.29 ** | 0.97 | 1.14 | 1.06 | 1.17 * |
| Education (1 = secondary or lower, 2 = tertiary) | 1.38 *** | 1.05 | 1.04 | 0.97 | 1.41 *** |
| Anticipatory solastalgia | 1.56 *** | 1.18 *** | 1.50 *** | 1.22 *** | 1.44 *** |
| R^2^ (Nagelkerke) | .31 | .05 | .21 | .08 | .29 |
| **New Zealand** | **Raising fuel tax** | **Expanding public transport** | **Increasing use of sustainable energy** | **Protecting forested and land areas** | **Increasing taxes on carbon intense foods** |
| Age | 1.00 | 1.01 * | 1.01 * | 1.00 | 0.99 * |
| Gender | 1.47 *** | 0.92 | 0.94 | 0.68 *** | 1.50 *** |
| Residence (1 = rural, 2 = urban) | 0.94 | 1.10 | 0.97 | 1.15 | 0.77 ** |
| Education (1 = secondary or lower, 2 = tertiary) | 1.25 ** | 0.99 | 0.95 | 1.04 | 1.27 *** |
| Anticipatory solastalgia | 1.52 *** | 1.26 *** | 1.27 *** | 1.33 *** | 1.48 *** |
| R^2^ (Nagelkerke) | .20 | .07 | .07 | .12 | .20 |

*Note*. Effect sizes are odd ratios from probit ordinal regression analyses. Continuous predictors are unstandardized. **p* < 0.05, ***p* < 0.01, ****p* < 0.001

**Table S7.**

*Descriptive information, and correlation matrix for Australia (below the diagonal) and New Zealand (above the diagonal).*

|  | 1. | 2. | 3. | 4. | 5. | 6. | 7. | 8. |
| --- | --- | --- | --- | --- | --- | --- | --- | --- |
| 1. Helpless | - | .50 | .51 | .54 | .60 | .50 | .53 | .37 |
| 2. Angry | .59 | - | .56 | .64 | .60 | .51 | .55 | .42 |
| 3. Guilty | .63 | .65 | - | .70 | .60 | .50 | .53 | .42 |
| 4. Ashamed | .60 | .68 | .72 | - | .61 | .49 | .54 | .41 |
| 5. Depressed | .67 | .67 | .70 | .67 | - | .57 | .61 | .41 |
| 6. Pessimistic | .58 | .57 | .57 | .58 | .61 | - | .53 | .39 |
| 7. Anxious | .67 | .65 | .65 | .67 | .71 | .60 | - | .50 |
| 8. Anticipatory solastalgia | .46 | .46 | .45 | .46 | .48 | .41 | .54 | - |
| M(SD) Australia | 3.14 (1.31) | 3.09 (1.32) | 2.90 (1.34) | 2.96 (1.36) | 2.96 (1.33) | 3.11 (1.24) | 3.20 (1.29) | 5.24 (1.24) |
| M(SD) NZ | 3.29 (1.24) | 3.21 (1.27) | 3.02 (1.30) | 3.04 (1.29) | 2.99 (1.29) | 3.22 (1.18) | 3.36 (1.18) | 5.31 (1.09) |

*Note.* All correlations in Table S2 are significant at the *p* < .001 level.

**References**

Chen, F. F. (2007). Sensitivity of goodness of fit indexes to lack of measurement invariance. *Structural Equation Modeling: A Multidisciplinary Journal, 14*(3), 464-504.<https://doi.org/10.1080/10705510701301834>

Cologna, V., Mede, N. G., Berger, S., Besley, J. C., Brick, C., Joubert, M., et al. (2025, in press). Trust in scientists and their role in society across 68 countries. *Nature Human Behaviour,* <https://www.nature.com/articles/s41562-024-02090-5>

Faul, F., Erdfelder, E., Buchner, A., & Lang, A.-G. (2009). Statistical power analyzes using G*Power 3.1: Tests for correlation and regression analyses. *Behavior Research Methods, 41*, 1149-1160. <https://doi.org/10.3758/BRM.41.4.1149>

Kyriazos, T. A. (2018). Applied psychometrics: sample size and sample power considerations in factor analysis (EFA, CFA) and SEM in general. *Psychology, 9*(08), 2207. <https://doi.org/10.4236/psych.2018.98126>

1. Data quality measures included screening out participants who failed either of two instructional attention checks. These checks were performed on the full sample of 4465 Australians and 1873 New Zealanders (note that only a subset completed our measures). In the first attention check, participants were asked to write the number “213” in a text box and were removed if they failed to do so (n = 115 in Australia, n = 43 in NZ); in the second, they were asked to select the response option “strongly disagree” and were removed if they chose a different answer option (n = 813 in Australia, n = 318 in NZ). We also removed duplicate responses based on participants’ Bilendi & Respondi participant identification number (n = 12 in Australia, n = 8 in NZ). [↑](#footnote-ref-1)
